# Enhancing Patient Safety Education: Cross-Cultural Validation of the APSQ-III in Brazilian Healthcare Students

**DOI:** 10.3390/nursrep15020033

**Published:** 2025-01-23

**Authors:** João Daniel de Souza Menezes, Matheus Querino da Silva, Emerson Roberto dos Santos, Rodrigo Soares Ribeiro, Natália Almeida de Arnaldo Silva Rodriguez Castro, Isabela Amaral de Almeida Bistafa, Alex Bertolazzo Quitério, Eliana Fazuoli Chubaci, Sônia Maria Maciel Lopes, Flávia Cristina Custódio, Stela Regina Pedroso Vilela Torres de Carvalho, Gustavo Schiavinato, Thalissa Catricala, José Nathan Fernandes Rocha, Vânia Maria Sabadoto Brienze, Josimerci Ittavo Lamana Faria, Denise Cristina Mós Vaz Oliani, Antônio Hélio Oliani, Vânia Zaqueu Brandão, Júlio Cesar André, Rita de Cassia Helú Mendonça Ribeiro

**Affiliations:** 1 Center for Studies and Development of Health Education, Faculty of Medicine of São José do Rio Preto (CEDES/FAMERP), São José do Rio Preto 15090-000, Brazil; matheusquirino@hotmail.com (M.Q.d.S.); emerson.santos@edu.famerp.br (E.R.d.S.); natalia.castro@edu.famerp.br (N.A.d.A.S.R.C.); isabela.bistafa@edu.famerp.br (I.A.d.A.B.); alex.quiterio@edu.famerp.br (A.B.Q.); eliana.chubaci@edu.famerp.br (E.F.C.); sonia.lopes@edu.famerp.br (S.M.M.L.); fccustodio@funecsantafe.edu.br (F.C.C.); stelarpvs@hotmail.com (S.R.P.V.T.d.C.); gustavo.schiavinato@edu.famerp.br (G.S.); thacatricala@hotmail.com (T.C.); nathan_fisioterapia@hotmail.com (J.N.F.R.); vania.brienze@hospitaldebase.com.br (V.M.S.B.); josimerci.faria@edu.famerp.br (J.I.L.F.); vaniazaqueu@famerp.br (V.Z.B.); julio.andre@edu.famerp.br (J.C.A.); 2 Postgraduate Department, Faculty of Medicine of São José do Rio Preto, Av. Brg. Faria Lima, 5416, Vila Sao Pedro, São José do Rio Preto 15090-000, Brazil; merodrigoribi@gmail.com (R.S.R.); ritadecassia@famerp.br (R.d.C.H.M.R.); 3 University Hospital Center Cova da Beira, University of Beira Interior, 6200-251 Covilhã, Portugal; vaz.oliani@gmail.com (D.C.M.V.O.); oliani@famerp.br (A.H.O.)

**Keywords:** patient safety, attitudes, medical student, nursing students, cross-cultural adaptation, validation, psychometrics

## Abstract

**Background:** This study aimed to adapt and validate the Attitudes to Patient Safety Questionnaire (APSQ-III) for Brazilian Portuguese and to compare patient safety attitudes between medical and nursing students. Given the critical role of assessing safety attitudes in shaping future healthcare professionals, this research addresses a significant gap in the Brazilian educational context. **Materials and Methods:** The cross-cultural adaptation process adhered to the guidelines of for the Process of Cross-Cultural Adaptation of Self-Report Measures, encompassing translation, synthesis, back-translation, and expert committee evaluation. The adapted APSQ-III was administered to a sample of 423 undergraduate students from medicine and nursing courses. Confirmatory factor analysis (CFA) was conducted to verify the factor structure, while reliability was assessed using Cronbach’s alpha, McDonald’s omega, and composite reliability measures. **Results:** The CFA supported an acceptable fit for the nine-factor model with 26 items, following the exclusion of 4 items (χ^2^/df = 1.92; CFI = 0.90; TLI = 0.89; RMSEA = 0.05; SRMR = 0.07). Factor loadings ranged from 0.30 to 0.82, with satisfactory reliability indices, except for factors 4 (α = 0.47; ω = 0.48) and 9 (α = 0.54; ω = 0.54). Significant differences were discovered between medical and nursing students in four factors, and gender differences were noted in five items, highlighting the diverse perceptions of patient safety across these groups. **Conclusions:** The Brazilian version of the APSQ-III demonstrated adequate validity and reliability for seven out of the nine original factors. It is recommended to use the scale with modifications, such as developing a reduced version excluding factors with low reliability, to enhance its applicability. This study contributes to advancing patient safety research and education in Brazil, providing a robust tool for evaluating and improving safety attitudes among healthcare students. Future research should focus on refining the instrument and exploring its application in diverse healthcare educational settings across Brazil.

## 1. Introduction

In the field of patient safety, assessing the knowledge and attitudes of healthcare students is crucial for identifying areas for improvement in patient safety education and for monitoring changes in attitudes over time [1]. The Attitudes to Patient Safety Questionnaire (APSQ) is a widely used instrument developed to measure attitudes towards patient safety among medical students and tutors [1]. The APSQ has undergone several revisions, with the most recent version being the APSQ-III, which consists of 26 items distributed across nine factors [1]. Understanding these attitudes is particularly important as they can influence the behavior of future healthcare professionals and impact patient safety outcomes [2].

Patient safety has gained increasing attention in Brazil in recent years with the implementation of public policies and educational initiatives aimed at improving healthcare quality and safety [3]. The Brazilian National Patient Safety Program, established in 2013, emphasizes the importance of integrating patient safety concepts into healthcare education curricula [4]. However, despite these advancements, there remains a significant gap in the systematic assessment of patient safety attitudes among healthcare students in Brazil.

The adaptation and validation of psychological instruments in different languages and cultures are essential to ensure their applicability and relevance in diverse contexts [5]. Cross-cultural adaptation involves not only the translation of the instrument but also the consideration of cultural differences and the necessary adjustments to maintain the instrument’s validity [6]. Previous studies have used the APSQ in different countries, such as the United Kingdom [1], Australia [7], and Saudi Arabia [8], demonstrating its applicability in various cultural contexts. Recent validations of the APSQ-III in countries like China and Turkey further underscore its cross-cultural relevance and utility in diverse healthcare settings [9,10].

Considering the relevance of the APSQ-III and the need for validated instruments to assess attitudes towards patient safety in the Brazilian context, this study aims to perform the cross-cultural adaptation and validation of the APSQ-III for the Portuguese language and Brazilian culture. The choice of APSQ-III for adaptation is based on its comprehensive coverage of patient safety domains, robust psychometric properties, and widespread use in international research, making it particularly suitable for cross-cultural comparisons [11].

The availability of an adapted and validated version of the APSQ-III will allow the assessment of attitudes and knowledge about patient safety in incoming students of healthcare courses in Brazil, providing subsidies for the improvement of patient safety education and the monitoring of changes in attitudes throughout professional training. Moreover, this study will contribute to the growing body of literature on patient safety education in different cultural contexts, enabling comparisons between Brazilian healthcare students and their international counterparts [12]. Recent studies have highlighted the importance of such assessments in enhancing patient safety education and practices [7,13].

Additionally, this research aims to explore potential differences in patient safety attitudes between medical and nursing students, as previous studies have suggested variations in safety culture perceptions across healthcare disciplines [14]. Understanding these differences can inform the development of tailored educational interventions and promote interprofessional collaboration in patient safety initiatives. This aligns with recent findings on the effectiveness of interprofessional education in improving patient safety outcomes [2,15].

By providing a validated Brazilian Portuguese version of the APSQ-III, this study not only fills a methodological gap but also contributes to advancing patient safety education in Brazil. The specific objectives of this study are (1) to conduct the cross-cultural adaptation of the APSQ-III to Brazilian Portuguese; (2) to evaluate the psychometric properties of the adapted version; and (3) to compare patient safety attitudes between medical and nursing students in Brazil.

## 2. Materials and Methods

### 2.1. Participants and Sample Size

The study included a convenience sample of incoming undergraduate students from healthcare courses, including medicine, nursing, and psychology, from a Brazilian public university. The sample size was determined based on guidelines for validation studies and considered a minimum of 10 participants per instrument item [13], resulting in 300. The final sample consisted of 423 students (50% medicine, 50% nursing), with ages ranging from 18 to 35 years (mean = 26.5, SD = 4.91).

Students regularly enrolled in the selected courses who agreed to participate voluntarily in the study were included. There were no exclusions among those who agreed to participate in the study since the form did not allow submission without complete filling of the instruments. The response rate was 84.6% (423 out of 500 invited students).

#### Inclusion and Exclusion Criteria

Inclusion criteria:

- Regularly enrolled undergraduate students in medicine, nursing, or psychology courses at the participating Brazilian public university;
- Age 18 years or older;
- Willingness to voluntarily participate in the study.

Exclusion criteria:

- Students not regularly enrolled in the selected healthcare courses;
- Incomplete submission of the study instruments;
- Inability to provide informed consent.

No exclusions were made among those who agreed to participate and completed the survey, as the online form was designed to prevent submission without complete filling of all required fields.

### 2.2. Instruments

The instrument to be adapted and validated was the Attitudes to Patient Safety Questionnaire III (APSQ-III), developed by [1]. The APSQ-III consists of 26 items, distributed across nine dimensions: (1) Teamwork, (2) Error and responsibility, (3) Confidence, (4) Training and skills, (5) Error reporting, (6) Organizational factors, (7) Individual factors, (8) Patient factors, and (9) Error disclosure and discussion. The items are answered on a 5-point Likert scale, ranging from “strongly disagree” to “strongly agree”. In Appendix A, we provide a comprehensive list of the original and translated items of the APSQ-III.

To assess convergent validity, the Safety Attitudes Scale (EAS) [15] was administered alongside the APSQ-III. Additionally, a sociodemographic questionnaire, developed by the researchers, was used to collect relevant participant information and characterize the sample.

### 2.3. Procedures

The cross-cultural adaptation process of the APSQ-III followed the guidelines proposed by Beaton et al. (2000) [16] and was carried out in two phases:

Phase 1 of the cross-cultural adaptation process encompassed translation, synthesis, back-translation, and expert committee evaluation processes. This phase was conducted as follows:

1. Translation: Three independent translations were performed by native Brazilian Portuguese translators with expertise in healthcare terminology.
2. Synthesis: The research team synthesized these translations into a single version, resolving discrepancies through consensus discussions. When consensus could not be reached, external linguistic experts were consulted.
3. Back-translation: Three native English translators, who had no prior exposure to the original questionnaire, independently back-translated the synthesized version.
4. Expert Committee Evaluation: An expert committee, comprising 5 professors from undergraduate healthcare courses and 3 specialists in cross-cultural adaptation, evaluated the proposed synthesis. They assessed semantic, idiomatic, experiential, and cultural equivalence using a structured evaluation form.
5. Resolution of Discrepancies: The committee discussed each item where equivalence was not achieved. Discrepancies were resolved through the following process:
  - Each committee member presented their rationale for their evaluation.
  - Alternative translations were proposed and discussed.
  - When necessary, the original authors of the APSQ-III were consulted for clarification.
  - Consensus was reached through iterative discussions and voting when required.
6. Pre-final Version: This rigorous process resulted in a pre-final version that achieved a consensus greater than 90% among the expert committee members for all items.

This systematic approach to resolving discrepancies during the cross-cultural adaptation process ensured a high degree of equivalence between the original and adapted versions of the APSQ-III, enhancing the instrument’s validity in the Brazilian context.

Phase 2 involved a pre-test with 50 students from the second and third years of the selected courses. Participants completed the pre-final version of the APSQ-III and were interviewed to identify potential issues and suggest changes before the final validation.

### 2.4. Data Collection

Data collection was conducted online from March 2023 to November 2023. Participants were recruited through the institution’s official email and provided informed consent prior to participation.

The study was approved by the institution’s Research Ethics Committee (Opinion 4,543,158, dated 17 February 2021), and all participants signed an Informed Consent Form.

### 2.5. Statistical Analysis

The psychometric properties of the adapted APSQ-III were evaluated through statistical analyses. The factor structure was verified through confirmatory factor analysis (CFA), using a polychoric correlation matrix and the Weighted Least Squares Mean and Variance Adjusted (WLSMV) estimation method, considered ideal for ordinal data [17]. This procedure is in line with the studies by Green et al. (2022) [18] and Stefanek et al. (2023) [19].

Model fit was assessed using three indices: the Root Mean Square Error of Approximation (RMSEA), Comparative Fit Index (CFI), and Tucker–Lewis Index (TLI). Following the recommendations of the psychometric literature [20,21,22,23], RMSEA values < 0.08, with a confidence interval not reaching 0.10, and CFI and TLI values ≥ 0.90 were considered acceptable for model retention. These cut-off values were chosen based on their widespread use in psychometric literature and their ability to balance sensitivity and specificity in model evaluation [24].

For the models considered acceptable, Cronbach’s alpha, McDonald’s omega, and composite reliability were calculated to verify the reliability of the latent variables. A latent variable was considered reliable if it presented a minimum value of 0.60 in McDonald’s omega or composite reliability.

To examine convergent validity, Pearson correlations were calculated between APSQ-III scores and the Safety Attitudes Scale (EAS) scores. Discriminant validity was assessed by comparing APSQ-III scores across different healthcare disciplines using one-way ANOVA.

Measurement invariance analysis was conducted to evaluate whether the APSQ-III functions similarly across different groups (e.g., medicine vs. nursing students). This analysis involved a series of increasingly constrained multi-group CFA models, as outlined by Putnick and Bornstein (2016) [24].

Confirmatory factor analyses were performed using the lavaan 0.6–8 package [25] of the R Statistical language (version 4.3.2) [26]. Reliability indices were calculated using the semTools package [27].

All analyses were conducted with the aim of rigorously evaluating the psychometric properties of the Brazilian Portuguese version of the APSQ-III, including its factor structure, reliability, and validity. The results of these analyses provided a comprehensive assessment of the instrument’s suitability for use in the Brazilian context and its potential for cross-cultural comparisons.

## 3. Results

### 3.1. Sample Characteristics

The sample consisted of 423 students, with 281 (66.43%) females and 142 (33.57%) males. Regarding age, 58.39% were between 18 and 20 years old, 39.48% were between 21 and 30 years old, and 2.13% were above 30 years old. This demographic distribution highlights the youthful nature of the cohort, predominantly composed of early-stage healthcare students.

### 3.2. Cross-Cultural Adaptation Results

During the cross-cultural adaptation process, several items required significant adjustments to ensure cultural equivalence. Specifically, items 7, 14, 22, and 30 were excluded following expert committee reviews due to difficulties in achieving semantic and cultural relevance. The adaptation process involved reaching a consensus over multiple rounds of evaluation, underscoring the importance of contextual sensitivity in instrument adaptation.

### 3.3. Confirmatory Factor Analysis

The confirmatory factor analysis supports a nine-factor model with 26 items, after excluding 4 items from the original scale. Key findings include

- Model fit indices: χ^2^/df = 1.92; CFI = 0.90; TLI = 0.89; RMSEA = 0.05; SRMR = 0.07;
- Factor loadings ranged from 0.30 to 0.82.

These results indicate an acceptable fit of the adapted APSQ-III to the data, supporting its structural validity in the Brazilian context—Table 1.

The model fit indices provide strong support for the validity of the nine-factor structure in the Brazilian context. The RMSEA value of 0.05 indicates a close fit, as it is below the recommended threshold of 0.08. The CFI value of 0.90 meets the acceptable threshold, suggesting a good fit between the hypothesized model and the observed data. While the TLI value of 0.89 is slightly below the conventional cutoff of 0.90, it is very close and, when considered alongside the other indices, still supports an acceptable model fit. The SRMR value of 0.07 is below the recommended maximum of 0.08, further confirming the model’s adequacy.

These fit indices collectively demonstrate that the adapted APSQ-III maintains its construct validity in the Brazilian context. The close alignment with established psychometric standards indicates that the instrument is effectively capturing the intended patient safety attitude constructs among Brazilian healthcare students. This provides confidence in the use of the adapted APSQ-III for assessing and comparing patient safety attitudes in this population.

It’s noteworthy that this acceptable model fit was achieved after the exclusion of four items. This suggests that the removal of these items enhanced the instrument’s cultural and semantic relevance without compromising its overall structural integrity. The improved model fit following these exclusions underscores the importance of cultural adaptation in maintaining an instrument’s validity across different contexts.

While the overall model fit is acceptable, the slightly lower TLI value and the reliability issues in factors 4 and 9 indicate areas for potential refinement in future iterations of the Brazilian APSQ-III. Further research could explore whether additional modifications or alternative factor structures might yield even better fit indices, potentially enhancing the instrument’s validity and reliability in the Brazilian healthcare education context.

### 3.4. Reliability Analysis

Reliability indices of the factors were satisfactory, except for factors 4 and 9, with Cronbach’s alpha and McDonald’s omega values below the acceptable threshold (α = 0.47; ω = 0.48 for F4 and α = 0.54; ω = 0.54 for F9). These findings suggest potential areas for refinement in these factors, possibly indicating cultural nuances that were not fully captured during adaptation.

The lower reliability of factors F4 (Inevitability of error) and F9 (Importance of patient safety in the curriculum) may reflect cultural nuances in the Brazilian healthcare education context. In Brazil, the concept of error inevitability might be perceived differently due to the country’s recent emphasis on patient safety initiatives. Similarly, the importance of patient safety in curricula may be undergoing a transition phase as educational institutions adapt to new national guidelines, potentially leading to varied interpretations among students.

### 3.5. Inter-Group Comparisons

In the analysis of patient safety attitudes between medical and nursing students, significant differences were identified in four key factors:

1. Patient safety training received (F1): Medical students showed more positive attitudes towards the training they received in patient safety compared to nursing students. This suggests that medical curricula may be more effective in providing patient safety education or that medical students perceive such training as more integral to their education.
2. Confidence in reporting errors (F2): Medical students demonstrated greater confidence in reporting errors than their nursing counterparts. This could be indicative of a cultural or educational emphasis on error reporting within medical training programs, highlighting the importance of transparency and accountability in medical practice.
3. Professional incompetence as a cause of error (F5): Nursing students had a higher perception that professional incompetence is a significant cause of errors. This perception might reflect differences in training emphasis or experiential learning opportunities between the two fields.
4. Importance of patient safety in the curriculum (F9): Medical students also rated the importance of patient safety within their curriculum more positively than nursing students. This disparity might suggest a need for nursing programs to more explicitly integrate patient safety into their core educational objectives.

These observed differences between medical and nursing students in their attitudes towards patient safety reflect potential disparities in curricular emphasis and professional culture. The more positive attitudes of medical students towards patient safety training (F1) and the importance of patient safety in the curriculum (F9) suggest that medical education in Brazil may have more explicitly integrated these concepts into their programs. This could be a result of recent reforms in medical education that have placed greater emphasis on patient safety.

The greater confidence among medical students in reporting errors (F2) is particularly noteworthy. This difference might be attributed to several factors:

1. Medical curricula may include more explicit training on error-reporting processes.
2. The hierarchical structure in healthcare settings might empower medical students to feel more confident in reporting errors.
3. There may be cultural differences in how responsibility and accountability are perceived in medical versus nursing education.

**Figure 1:**
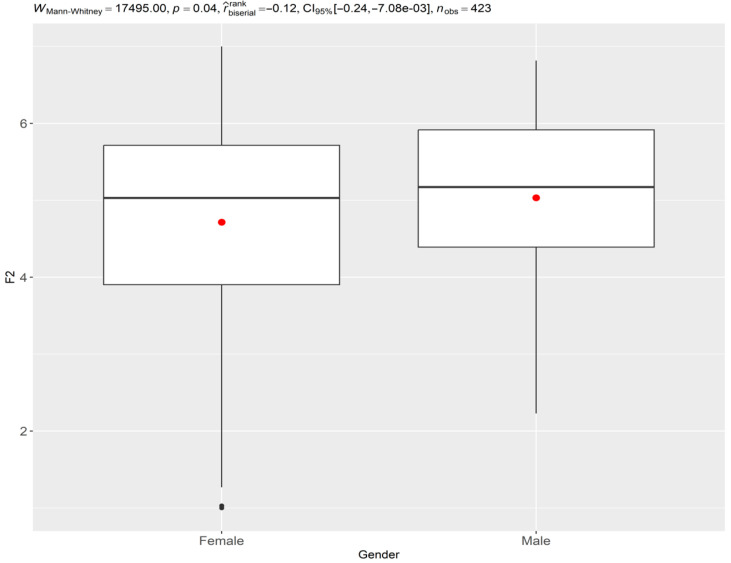
Comparison of confidence in reporting errors (F2) by gender. Source: Author.

Significant gender differences were noted in 5 of the 26 items analyzed (Table 2):

- Men (male) exhibited greater confidence in reporting errors (items 4, 5, and 6). This finding suggests a potential gender-based difference in attitudes toward error disclosure, which may be influenced by confidence levels or social and educational experiences—Figure 1.
- Women (female) were slightly more likely to perceive errors as being caused by carelessness or professional incompetence (items 15 and 18). This perception could be influenced by differing educational experiences or cultural expectations regarding accountability and error prevention—Figure 1.

These gender differences observed in error-reporting confidence and perceptions of error causation highlight important considerations for patient safety education. The higher confidence in error reporting among male students may reflect broader societal gender norms or differences in professional socialization processes. Conversely, the tendency for female students to attribute errors more to carelessness or incompetence suggests a potentially higher self-critical stance or different internalization of professional standards.

These findings underscore the need for patient safety education to address not only professional differences but also potential gender-based variations in attitudes and perceptions. Tailored educational interventions that consider these differences could enhance the effectiveness of patient safety training across all student groups.

### 3.6. Limitations in Invariance Analysis

Due to the absence of responses in all response categories for all items in both gender groups, it was not possible to perform the invariance analysis, an essential requirement for valid cross-group comparisons [24]. This limitation highlights the challenge of achieving comprehensive response coverage across diverse demographic groups, suggesting a need for further investigation into response patterns.

These results, while illuminating important differences across professional tracks and genders, also point to the complex interplay of cultural, educational, and social factors in shaping patient safety attitudes among healthcare students in Brazil. Further research is needed to explore the underlying causes of these differences and their implications for patient safety education and practice.

#### Guidelines and Standards Statement

This study was conducted in accordance with the STROBE (Strengthening the Reporting of Observational Studies in Epidemiology) guidelines for cross-sectional studies [28]. The cross-cultural adaptation process followed the guidelines proposed by Beaton et al. (2000) [16] for the cross-cultural adaptation of self-report measures. The psychometric validation procedures adhered to the standards outlined in the Standards for Educational and Psychological Testing [29].

## 4. Discussion

The present study aimed to adapt and validate the Attitudes to Patient Safety Questionnaire (APSQ-III) for Brazilian Portuguese and compare patient safety attitudes among medical and nursing students. The results indicated that the Brazilian version of the APSQ-III showed adequate evidence of internal structure validity and reliability for seven of the nine original factors, after the exclusion of four items. Furthermore, significant differences were found between genders only in the factor ‘Confidence in reporting errors’ (F2).

The confirmatory factor analysis indicated that the model with nine factors and 26 items showed an acceptable fit to the data, after the exclusion of items 7, 14, 22, and 30. These results are partially consistent with previous validation studies of the APSQ-III in other cultural contexts [30,31,32]. While the exclusion of these four items was necessary to ensure cultural and semantic relevance in the Brazilian context, we acknowledge that this modification may impact the instrument’s cross-cultural comparability. However, we believe that maintaining the integrity of the construct within the Brazilian cultural context takes precedence over strict adherence to the original structure. Future cross-cultural studies using this adapted version should take these modifications into account when making comparisons with other cultural contexts. However, the factors ‘Inevitability of error’ (F4) and ‘Importance of patient safety in the curriculum’ (F9) showed reliability below the acceptable level in the Brazilian sample, suggesting that these factors may not be suitable for use in this context [33,34,35]. The confirmation of the nine-factor structure in the Brazilian version suggests that the dimensions of patient safety attitudes are consistent across cultures. However, the differences observed in factors such as ‘Patient safety training received’ (F1) and ‘Professional incompetence as a cause of error’ (F5) between medical and nursing students may reflect curricular differences or professional culture within these courses in Brazil.

However, the reliability issues observed in factors F4 (Inevitability of error) and F9 (Importance of patient safety in the curriculum) warrant careful consideration. These lower reliability scores may impact the interpretation of results related to these specific dimensions. For instance, the concept of error inevitability (F4) might be perceived differently in the Brazilian healthcare context, possibly due to cultural factors or recent emphasis on error-prevention strategies. Similarly, the varied interpretations of the importance of patient safety in curricula (F9) could reflect the ongoing transition in educational approaches across different healthcare programs in Brazil. Future research should focus on refining these factors to better capture these concepts within the Brazilian context, possibly through qualitative studies to understand local interpretations of these constructs [36].

The findings of this study are particularly relevant in the Brazilian context, where recent patient safety initiatives, such as the National Patient Safety Program, emphasize the importance of integrating patient safety education into healthcare training. The validation of the APSQ-III provides a valuable tool for assessing the impact of these initiatives, offering insights into how educational programs can be tailored to enhance safety culture among healthcare students.

These findings have important implications for the application of the APSQ-III in Brazil. It is recommended that factors F4 and F9 not be used in their current form due to low reliability. A possible solution would be the creation of a reduced version of the APSQ-III in Portuguese, excluding problematic items and factors with low reliability [24,31,37]. This reduced version would still allow comparisons with international results while maintaining most of the original structure of the scale.

In the comparison between genders, only the factor ‘Confidence in reporting errors’ (F2) showed a statistically significant difference, with men showing greater confidence in reporting errors than women. This finding is consistent with previous studies that identified gender differences in patient safety attitudes [34,35,38]. However, it is important to note that this difference was found without performing an invariance analysis, which limits the interpretation of the results [24,39], since without this analysis, comparisons between groups may be biased, as it cannot be guaranteed that the instrument is measuring the construct of interest in the same way in both groups [40].

While our findings indicate notable gender-based differences in patient safety attitudes, particularly in error-reporting confidence and perceptions of error causation, it is crucial to interpret these results cautiously. The lack of measurement invariance analysis, due to the absence of responses in all categories for all items across gender groups, limits our ability to draw definitive conclusions about these differences. This limitation highlights a significant area for future research. Subsequent studies should aim to achieve more comprehensive response coverage across demographic groups and conduct rigorous measurement invariance analyses to validate these gender-based differences [24]. Such analyses would provide a more robust foundation for developing targeted interventions that address gender-specific needs in patient safety education.

Our findings partially align with previous validation studies of the APSQ-III conducted in different cultural contexts [30,31,32]. While some factors showed consistency, others, such as F4 and F9, did not meet reliability thresholds, diverging from results in countries like Australia and Saudi Arabia, where these factors were more robust [33,34,35]. The factors ’Inevitability of error’ (F4) and ’Importance of patient safety in the curriculum’ (F9) showed Cronbach’s alpha and McDonald’s omega values below the acceptable threshold (α = 0.47; ω = 0.48 for F4 and α = 0.54; ω = 0.54 for F9). These results indicate insufficient reliability of these factors in the Brazilian context. This may suggest that these concepts are understood or operationalized differently in Brazilian healthcare culture compared to the instrument’s original context. Future qualitative studies are needed to explore how these concepts are interpreted by Brazilian healthcare professionals in training, which may lead to a reformulation of these items or the development of new, more culturally appropriate items to capture these dimensions in the Brazilian context. Our findings on gender differences in patient safety attitudes contrast with the results of Østergaard et al. (2022) [41] in Denmark but are consistent with the study by Lim et al. (2023) [42] in China. This suggests that cultural factors may influence how gender relates to patient safety attitudes, highlighting the need for culturally sensitive educational strategies.

Our study provides the first validated Brazilian Portuguese version of the APSQ-III, adding to the growing body of international adaptations of this instrument. While our results show some consistency with previous validations [30,31,32], there are notable differences that warrant discussion.

For instance, the Chinese version by Huang et al. (2022) [9] retained all the original items and showed good reliability across all factors, contrasting with our findings of low reliability in factors F4 and F9. The Turkish adaptation by Taskiran et al. (2020) [10] also maintained the original structure but reported challenges with certain items, similar to our experience.

These variations highlight the complex interplay between cultural contexts and patient safety attitudes. The differences in factor reliability and item retention across adaptations suggest that patient safety concepts may be understood and operationalized differently across cultures. For example, our challenges with factors related to ’Inevitability of error’ (F4) and ’Importance of patient safety in the curriculum’ (F9) may reflect unique aspects of the Brazilian healthcare education system or cultural perceptions of error and safety.

Future research should aim to conduct more rigorous cross-cultural analyses, potentially using multi-group confirmatory factor analysis to directly compare factor structures across different cultural adaptations. This would provide valuable insights into how patient safety attitudes vary globally and inform the development of culturally sensitive, yet internationally comparable, patient safety education strategies.

The present study has some limitations, such as the sample composed only of medical and nursing students from a single institution and the cross-sectional design [30,33,34]. Despite these limitations, this study brings relevant contributions to the field of patient safety in Brazil, providing a useful tool for assessing patient safety attitudes in medical and nursing students [31,37,38].

However, it is important to note that this difference was found without performing an invariance analysis, which limits the interpretation of the results [24,39]. Due to the absence of responses in all categories for all items across gender groups, it was not possible to conduct measurement invariance analysis. Without this analysis, comparisons between groups may be biased, as it cannot be guaranteed that the instrument is measuring the construct of interest in the same way in both groups [40]. This limitation highlights a significant area for future research, where studies should aim to achieve more comprehensive response coverage across demographic groups and conduct rigorous measurement invariance analyses to validate these gender-based differences.

The inability to conduct measurement invariance analysis due to response pattern limitations is a significant constraint of this study. This limitation affects our capacity to make definitive comparisons between groups, particularly in terms of gender differences. Future research should employ strategies to ensure more uniform response distributions across all items and demographic groups. This might involve larger, more diverse samples or the use of mixed-methods approaches to capture a wider range of perspectives [43].

The results of this study are particularly relevant in the Brazilian context, where recent patient safety initiatives, such as the National Patient Safety Program, emphasize the integration of safety education into healthcare training. The validated APSQ-III offers a valuable tool for assessing the impact of these initiatives and guiding curriculum development. Educational institutions can leverage these insights to enhance areas where students exhibit less positive attitudes, such as specific dimensions of patient safety. This could involve developing targeted educational interventions that address identified gaps and promote a culture of safety.

## 5. Conclusions

This study successfully adapted and validated the Attitudes to Patient Safety Questionnaire (APSQ-III) for Brazilian Portuguese, providing a significant advancement in the assessment of patient safety attitudes among healthcare students in Brazil. By achieving a reliable and culturally relevant version of the APSQ-III, this research fills a critical methodological gap and enhances the tools available for evaluating patient safety education in diverse contexts.

To address the low-reliability factors identified in this study, we propose the development of a reduced version of the APSQ-III specifically tailored for the Brazilian context. This adapted version would exclude problematic items and factors with low reliability while maintaining the core structure that allows for international comparisons.

The findings confirm the cross-cultural applicability of the APSQ-III, demonstrating that the core dimensions of patient safety attitudes are consistent across different cultural settings. This validation not only supports international comparisons but also highlights specific areas in Brazilian healthcare education that require attention, such as the integration of patient safety into nursing curricula and addressing gender-based differences in attitudes toward error reporting.

Despite the study’s limitations, including the single-institution sample and the cross-sectional design, it offers valuable insights into the educational needs of healthcare students. The identification of differences in attitudes between medical and nursing students underscores the importance of tailored educational interventions and interprofessional collaboration to foster a robust culture of patient safety.

We recommend that healthcare education programs in Brazil integrate the validated APSQ-III into their curriculum assessment processes. This integration would allow for regular evaluation of patient safety attitudes among students and enable data-driven improvements to patient safety education. Furthermore, based on the gender differences and variations between medical and nursing students identified in this study, we recommend the development of targeted educational interventions. These interventions should address specific areas where attitudes were found to be less positive, with the aim of fostering a more consistent and comprehensive patient safety culture across all healthcare professions.

Future research should aim to expand the scope of this study by incorporating longitudinal designs and diverse educational settings, allowing for a more comprehensive understanding of how patient safety attitudes evolve and impact professional practice.

To further validate and refine the APSQ-III in the Brazilian context, we propose establishing a national network of healthcare education institutions. This collaboration would facilitate larger-scale studies, allowing for more robust analyses of regional variations and the impact of different educational approaches on patient safety attitudes.

Additionally, exploring the relationship between these attitudes and actual safety behaviors in clinical environments will be essential for translating educational improvements into tangible patient safety outcomes.

Overall, the validated Brazilian Portuguese version of the APSQ-III serves as a pivotal tool for educators, policymakers, and researchers, facilitating evidence-based curriculum development and the evaluation of educational interventions aimed at enhancing patient safety in Brazil. This study not only contributes to the academic literature on patient safety but also supports ongoing efforts to improve healthcare quality and safety on a global scale.

## 6. Limitations and Contributions of the Study

### 6.1. Contributions

This study offers several significant contributions to the field of patient safety:

- First Validated Version of APSQ-III in Brazilian Portuguese: It provides the first validated version of the Attitudes to Patient Safety Questionnaire (APSQ-III) in Brazilian Portuguese, filling a crucial gap in assessing patient safety attitudes in the Brazilian context.
- Cross-Cultural Applicability: The study demonstrates the cross-cultural applicability of the APSQ-III, contributing to the international literature on evaluating patient safety attitudes. This validation underscores the versatility of the APSQ-III across diverse cultural settings.
- Interprofessional Curriculum Insights: By identifying differences in patient safety attitudes between medical and nursing students, the study offers valuable insights for developing interprofessional curricula that address distinct educational needs and perceptions.
- Validated Tool for Educational Interventions: The validated APSQ-III serves as a tool to evaluate the impact of educational interventions on patient safety in Brazil, facilitating the assessment and improvement of safety education programs.

### 6.2. Limitations

This study presents several limitations that should be considered:

- Limited Sample: Conducted in a single educational institution, the study’s findings may not be generalizable to other regions of Brazil or different educational contexts. This limitation highlights the need for broader studies involving multiple institutions. The limited sample from a single educational institution not only restricts the generalizability of our findings to other regions of Brazil but also potentially masks regional variations in patient safety attitudes. This underscores the critical need for multi-institutional studies that can capture a more comprehensive picture of patient safety attitudes across diverse Brazilian healthcare educational settings. Future research should aim to include a more diverse sample population that better reflects the broader student demographics across Brazil, considering factors such as regional differences, public versus private institutions, and varying levels of healthcare education programs. Such an approach would provide a more nuanced understanding of patient safety attitudes and potentially reveal important variations that could inform targeted educational interventions.
- Cross-Sectional Design: The cross-sectional nature of the study does not allow for inferences about changes in attitudes over time or throughout academic training. Longitudinal studies are necessary to understand attitude evolution. While our cross-sectional design provides a snapshot of patient safety attitudes, it limits our ability to understand how these attitudes develop and change over time. This constraint is particularly significant in the context of healthcare education, where attitudes may evolve substantially from the beginning of professional training through early career experiences.
- Self-Report Bias: As the APSQ-III is a self-report instrument, responses may be influenced by social desirability or other response biases, potentially affecting the accuracy of the reported attitudes. The reliance on self-reported data introduces potential biases, including social desirability bias, which may lead to overreporting of positive attitudes towards patient safety. Future research could mitigate these biases by incorporating objective measures of patient safety knowledge and behaviors, or by employing mixed-methods approaches that combine quantitative surveys with qualitative interviews or observational studies.
- Low Reliability in Some Factors: The factors ‘Inevitability of error’ (F4) and ‘Importance of patient safety in the curriculum’ (F9) showed low reliability, which may limit the interpretation of these specific aspects and suggest the need for further refinement. The low reliability observed in factors F4 and F9 not only limits the interpretation of these specific aspects but also raises questions about the cultural equivalence of these constructs in the Brazilian context. This highlights the need for qualitative research to explore how these concepts are understood and operationalized in Brazilian healthcare education.
- Lack of Convergent Validation: The study did not include additional measures to assess the convergent validity of the adapted instrument, which could enhance understanding of its construct validity. The absence of convergent validation limits our ability to fully establish the construct validity of the adapted APSQ-III. Future studies should include additional validated measures of patient safety attitudes or related constructs to provide a more comprehensive validation of the instrument in the Brazilian context.
- Absence of Measurement Invariance Analysis: The absence of measurement invariance analysis limits the interpretation of comparisons between courses and genders, as it remains uncertain whether the instrument measures constructs equivalently across these groups.

### 6.3. Future Directions

Despite these limitations, this study provides a solid foundation for future research. Future studies could address these limitations by using more diverse samples, employing longitudinal designs, and including objective behavioral measures of patient safety. Such research efforts would further enhance the understanding and application of patient safety attitudes across different educational and cultural settings.

To address the limitations identified in this study, future research should focus on:

1. Refining the factors with lower reliability (F4 and F9) through qualitative exploration of these concepts in the Brazilian healthcare education context.
2. Conducting comprehensive measurement invariance analyses with larger, more diverse samples to validate group comparisons, especially regarding gender differences.
3. Exploring the underlying causes of the observed differences between medical and nursing students’ attitudes towards patient safety, possibly through longitudinal studies that track changes in attitudes throughout their educational journey [44].
4. Investigating the impact of cultural and systemic factors on patient safety attitudes in Brazil, considering the country’s unique healthcare and educational landscape.

### 6.4. Actionable Recommendations for Curriculum Integration and Interventions

1. Tailored Curriculum Enhancement:
  - Revise medical and nursing curricula to address the identified gaps in patient safety attitudes, particularly focusing on areas where students showed less positive attitudes.
  - Develop specific modules addressing error-reporting confidence, emphasizing its importance in both medical and nursing practice.
2. Interprofessional Education Initiatives:
  - Implement joint training sessions for medical and nursing students to bridge the attitudinal differences observed in factors like ‘Patient safety training received’ (F1) and ‘Professional incompetence as a cause of error’ (F5).
  - Create case-based learning scenarios that require collaboration between medical and nursing students, fostering a shared understanding of patient safety concepts.
3. Gender-Sensitive Approaches:
  - Design targeted interventions to address gender differences in error reporting confidence, such as mentorship programs or specialized workshops that empower all students to report errors confidently.
4. Cultural Adaptation of Patient Safety Concepts:
  - Develop educational materials that contextualize patient safety concepts within the Brazilian healthcare system, addressing the cultural nuances identified in factors F4 (Inevitability of error) and F9 (Importance of patient safety in the curriculum).
  - Conduct focus groups with students and educators to refine the understanding and teaching of these concepts in the Brazilian context.
5. Practical Skill Development:
  - Incorporate simulation-based learning experiences that allow students to practice patient safety skills in a controlled environment, with scenarios tailored to common challenges in Brazilian healthcare settings.
6. Continuous Assessment and Curriculum Refinement:
  - Implement regular assessments using the adapted APSQ-III throughout students’ educational journeys to monitor changes in attitudes and adjust curricula accordingly.
  - Use these assessments to evaluate the effectiveness of new educational interventions and make data-driven improvements.
7. Faculty Development Programs:
  - Provide training for educators on effectively teaching patient safety concepts, emphasizing the cultural nuances and interprofessional aspects identified in this study.
  - Encourage faculty to integrate patient safety principles across various courses and clinical rotations.
8. Alignment with National Initiatives:
  - Develop curriculum components that directly address the goals of Brazil’s National Patient Safety Program, ensuring that students are prepared to contribute to national patient safety efforts upon graduation.
9. Research Integration:
  - Establish a research program that allows students to participate in patient safety research projects, fostering a culture of inquiry and evidence-based practice in patient safety.
10. Clinical Practice Integration:
  - Create clear guidelines for applying patient safety principles during clinical rotations, ensuring that theoretical knowledge is translated into practical skills.
  - Develop a system for students to report and reflect on patient safety incidents observed during their clinical experiences.

These recommendations aim to translate our research findings into practical strategies for enhancing patient safety education in Brazilian healthcare institutions, addressing the specific needs and cultural context identified in our study.

## Figures and Tables

**Table 1:** Factor loadings and reliability indices of the factors of the Brazilian version of the APSQ-III (*n* = 423, FAMERP, Brazil, 2023).

Factor	*n*	Mean	SD	Min	Max	Cronbach’s Alpha	McDonald’s Omega	Composite Reliability
F1	3	0.780	0.167	0.601	0.932	0.770	0.800	0.619
F2	3	0.793	0.122	0.705	0.933	0.790	0.820	0.636
F3	3	0.871	0.076	0.785	0.931	0.830	0.850	0.761
F4	3	0.596	0.328	0.231	0.867	0.310	0.330	0.382
F5	4	0.669	0.121	0.533	0.826	0.720	0.740	0.524
F6	3	0.761	0.083	0.688	0.852	0.700	0.740	0.582
F7	2	0.905	0.092	0.840	0.970	0.800	0.840	0.756
F8	2	0.840	0.131	0.747	0.932	0.750	0.780	0.621
F9	3	0.676	0.198	0.451	0.819	0.420	0.480	0.470

Note. *n*: Number of items per factor; SD: Standard deviation; Min: minimum; Max: Maximum. F1: Patient safety training received; F2: Confidence in reporting errors; F3: Working hours as a cause of error; F4: Inevitability of error; F5: Professional incompetence as a cause of error; F6: Responsibility for disclosure; F7: Team functioning; F8: Patient involvement in error reduction; F9: Importance of patient safety in the curriculum. Source: Author.

**Table 2:** Comparison of patient safety attitudes between gender. (*n* = 423, FAMERP, Brazil, 2023).

Factor	Group	*n*	Median [min, max]	W	*p*-Value	r-Biserial [IC 95%]
F1	Female	281	5.20 [1.00, 6.84]	18,531.00	0.23	−0.07 [−0.19, 0.05]
	Male	142	5.28 [1.69, 7.00]			
F2	Female	281	5.03 [1.00, 7.00]	17,495.00	0.04	−0.12 [−0.24, −0.01]
	Male	142	5.17 [2.23, 6.82]			
F3	Female	281	5.98 [1.33, 7.00]	19,317.00	0.59	−0.03 [−0.15, 0.08]
	Male	142	6.05 [1.37, 6.81]			
F4	Female	281	6.10 [3.00, 7.00]	18,738.00	0.31	−0.06 [−0.18, 0.06]
	Male	142	6.12 [3.54, 6.68]			
F5	Female	281	3.25 [1.14, 6.66]	19,595.00	0.76	−0.02 [−0.13, 0.10]
	Male	142	3.31 [1.00, 6.75]			
F6	Female	281	2.49 [1.00, 6.33]	20,350.00	0.74	0.02 [−0.10, 0.14]
	Male	142	2.54 [1.05, 5.95]			
F7	Female	281	6.32 [2.50, 6.89]	18,054.00	0.11	−0.10 [−0.21, 0.02]
	Male	142	6.35 [2.68, 7.00]			
F8	Female	281	5.38 [1.00, 6.84]	18,721.00	0.30	−0.06 [−0.18, 0.05]
	Male	142	5.47 [1.85, 7.00]			
F9	Female	281	6.30 [1.25, 6.87]	18,322.00	0.17	−0.08 [−0.20, 0.03]
	Male	142	6.38 [3.09, 7.00]			

Note. min: Minimum; max: Maximum; *n*: Sample size; F1: Patient safety training received; F2: Confidence when reporting error; F3: Working hours as cause of error; F4: Inevitability of error; F5: Professional incompetence as cause of error; F6: Responsibility for disclosure; F7: Team functioning; F8: Patient involvement in error reduction; F9: Importance of patient safety in the curriculum. Source: Author.

## Data Availability

The original contributions presented in this study are included in the article. Further inquiries can be directed to the corresponding author(s).

## Public Involvement Statement

According to GRIPP2, the involvement of undergraduates from the Sao Jose do Rio Preto Medical School was reactive (answering questionnaires and contributing perceptions), but the research did not include the public directly in the design, development, and analysis phases. Encouraging participation and preserving confidentiality reflect ethical efforts to ensure the comfort of the undergraduates without their active inclusion in the study design.

## Guidelines and Standards Statement

This manuscript was drafted against the Guidelines for the Process of Cross-Cultural Adaptation of Self-Report Measures for Cross-Cultural Adaptation research.

## Comparison Between the Original and Brazilian Versions of the APSQ-III

**Factor Code**	**Item Number**	**Item in Original Language**	**Item in Brazilian Portuguese**
F1	1	My training is preparing me to understand the causes of medical errors	Meu treinamento está me preparando para entender as causas dos erros médicos/de enfermagem.
	2	I have a good understanding of patient safety issues as a result of my undergraduate medical training	Tenho um bom entendimento das questões de segurança do paciente como resultado do meu treinamento médico/de enfermagem de graduação.
	3	My training is preparing me to prevent medical errors	Meu treinamento está me preparando para prevenir erros médicos de enfermagem.
F2	4	I would feel comfortable reporting any errors I had made, no matter how serious the outcome had been for the patient	Eu me sentiria confortável em relatar quaisquer erros que cometi, independentemente da gravidade do resultado para o paciente.
	5	I would feel comfortable reporting any errors other people had made, no matter how serious the outcome had been for the patient	Eu me sentiria confortável em relatar quaisquer erros que outras pessoas cometeram, independentemente da gravidade do resultado para o paciente.
	6	I am confident I could talk openly to my supervisor about an error I had made if it had resulted in potential or actual harm to my patient	Estou confiante de que poderia falar abertamente com meu supervisor sobre um erro que cometi se isso resultasse em dano potencial ou real ao meu paciente.
	7	I feel confident I could report an error I had made without feeling I would be blamed	Sinto-me confiante de que poderia relatar um erro que cometi sem sentir que seria culpado.
F3	8	Shorter shifts for doctors will reduce medical errors	Turnos mais curtos para os médicos/enfermeiros reduzirão os erros médicos/de enfermagem.
	9	By not taking regular breaks during shifts, doctors are at an increased risk of making errors	Ao não fazer pausas regulares durante os turnos, os médicos/enfermeiros correm um risco maior de cometer erros.
	10	The number of hours doctors work increases the likelihood of making medical errors	O número de horas que os médicos/enfermeiros trabalham aumenta a probabilidade de cometer erros médicos/de enfermagem.
F4	11	Even the most experienced and competent doctors make errors	Mesmo os médicos/enfermeiros mais experientes e competentes cometem erros.
	12	Human error is inevitable	O erro humano é inevitável.
	13	I don’t think I will make errors once I am a qualified doctor	Não acho que cometerei erros uma vez que eu seja um médico/enfermeiro qualificado.
F5	14	Most medical errors result from careless nurses	A maioria dos erros médicos/de enfermagem resulta de enfermeiros/médicos descuidados.
	15	If people paid more attention at work, medical errors would be avoided	Se as pessoas prestassem mais atenção no trabalho, os erros médicos/de enfermagem seriam evitados.
	16	Most medical errors result from careless doctors	A maioria dos erros médicos/ de enfermagem resulta de médicos/enfermeiros descuidados.
	17	Medical errors are a sign of incompetence	Erros médicos/de enfermagem são um sinal de incompetência.
F6	18	It is not necessary to report errors which do not result in adverse outcomes for the patient	Não é necessário relatar erros que não resultem em desfechos adversos para o paciente.
	19	Doctors have a responsibility to disclose errors to patients only if they result in patient harm	Os médicos/enfermeiros têm a responsabilidade de divulgar erros aos pacientes apenas se resultarem em dano ao paciente.
	20	All medical errors should be reported	Todos os erros médicos/de enfermagem devem ser relatados.
F7	21	Better multi-disciplinary teamwork will reduce medical errors	Melhor trabalho em equipe multidisciplinar reduzirá os erros médicos/. de enfermagem
	22	Teaching teamwork skills will reduce medical errors	Ensinar habilidades de trabalho em equipe reduzirá os erros médicos/de enfermagem.
F8	23	Patients have an important role in preventing medical errors	Os pacientes têm um papel importante na prevenção de erros médicos/de enfermagem.
	24	Encouraging patients to be more involved in their care can help to reduce the risk of medical errors occurring	Incentivar os pacientes a se envolverem mais em seu cuidado pode ajudar a reduzir o risco de ocorrência de erros médicos/de enfermagem.
F9	25	Teaching students about patient safety should be an important priority in medical students’ training	Ensinar aos alunos sobre segurança do paciente deve ser uma prioridade importante no treinamento de estudantes de medicina/enfermagem.
	26	Learning about patient safety issues before I qualify will enable me to become a more effective doctor	Aprender sobre questões de segurança do paciente antes de me qualificar me permitirá tornar-me um médico/enfermeiro mais eficaz.
